# Biochar Treatment Resulted in a Combined Effect on Soybean Growth Promotion and a Shift in Plant Growth Promoting Rhizobacteria

**DOI:** 10.3389/fmicb.2016.00209

**Published:** 2016-02-25

**Authors:** Dilfuza Egamberdieva, Stephan Wirth, Undine Behrendt, Elsayed F. Abd_Allah, Gabriele Berg

**Affiliations:** ^1^Institute for Landscape Biogeochemistry, Leibniz Centre for Agricultural Landscape ResearchMüncheberg, Germany; ^2^Plant Production Department, Faculty of Food and Agricultural Sciences, King Saud UniversityRiyadh, Saudi Arabia; ^3^Institute of Environmental Biotechnology, Graz University of TechnologyGraz, Austria

**Keywords:** soybean, rhizosphere, plant growth promoting rhizobacteria, biochar

## Abstract

The application of biochar to soil is considered to have the potential for long-term soil carbon sequestration, as well as for improving plant growth and suppressing soil pathogens. In our study we evaluated the effect of biochar on the plant growth of soybeans, as well as on the community composition of root-associated bacteria with plant growth promoting traits. Two types of biochar, namely, maize biochar (MBC), wood biochar (WBC), and hydrochar (HTC) were used for pot experiments to monitor plant growth. Soybean plants grown in soil amended with HTC char (2%) showed the best performance and were collected for isolation and further characterization of root-associated bacteria for multiple plant growth promoting traits. Only HTC char amendment resulted in a statistically significant increase in the root and shoot dry weight of soybeans. Interestingly, rhizosphere isolates from HTC char amended soil showed higher diversity than the rhizosphere isolates from the control soil. In addition, a higher proportion of isolates from HTC char amended soil compared with control soil was found to express plant growth promoting properties and showed antagonistic activity against one or more phytopathogenic fungi. Our study provided evidence that improved plant growth by biochar incorporation into soil results from the combination of a direct effect that is dependent on the type of char and a microbiome shift in root-associated beneficial bacteria.

## Introduction

Biochar is a fine-grained charcoal that is rich in organic carbon, produced by pyrolysis or by heating biomass in a low oxygen environment and has been used worldwide as a soil amendment to increase soil fertility (Lehmann and Joseph, 2009; Schomberg et al., 2012). However, biochar application is a very old method of improving soil quality and plant growth, as reported by the Amazonian Dark Earths (ADE) or Terra Preta de Índio formed in the past by pre-Columbian populations (Barbosa Lima et al., 2015). Presently, there are extensive literature reviews about the use of biochar and hydrochar to mitigate climate change by increasing carbon storage in soils (Lehmann et al., 2011). Other topics are about improving soil nutrient availability and the growth and development of agriculturally important crops, inducing systemic resistance in plants against soil borne fungal pathogens (Elad et al., 2010). Improvements in plant growth and yield following biochar application have been reported under field and greenhouse conditions for a variety of crops, including legumes such as soybean (*Glycine max* L.; Tagoe et al., 2008) and common bean (*Phaseolus vulgaris*; Rondon et al., 2007). Suppadit et al. (2012) reported an increased number of nodules, plant height, dry weight, yield and nutrient uptake in soybeans by quail litter biochar. Reibe et al. (2015a) observed that plant growth and development were affected by the type of char and rates of application, e.g., increasing amounts of fermented hydrochar (HTC) increased shoot biomass and the shoot/root ratio in case of spring wheat. Whereas the agricultural benefits of incorporating biochar into soils are frequently reported, there is little and incomplete evidence concerning the mechanisms of plant growth stimulation or the protection of plants from fungal pathogens by biochar. There are several studies explaining an indirect effect of biochar on root growth and development by altering soil properties, such as porosity and pore size distribution, water holding capacity, mechanical stability, sorption properties and the bioavailability of nutrients and trace elements (Laird et al., 2010; Spokas et al., 2010), but the functional response of soil microbial populations after biochar amendments are not well-understood (Lehmann et al., 2011). Anders et al. (2013) stated that the change in the structure of the microbial community by biochar application is an indirect effect and depends on soil nutrient status. Barbosa Lima et al. (2015) revealed that soil type contributes to the composition of bacterial communities in studies of forest sites (*Mimosa debilis*) and open areas (*Senna alata*) in the Amazon region. However, despite numerous reports on microbial changes induced by biochar application in various cropping systems, there have been no studies on how biochar affects the diversity and physiological activity of plant growth stimulating rhizobacteria, especially in legumes.

Most members of root-associated microbes are capable to promote plant growth and are commonly studied for their ability to stimulate plant yield, nutrient uptake, stress tolerance, and biological control of soil borne disease (Egamberdieva et al., 2008, 2011; Argaw, 2012; Berg et al., 2013a). The composition of rhizosphere bacteria is influenced not only by the plant species but also by the soil type (Berg and Smalla, 2009). The mechanisms involved in the beneficial effects conferred to plants include the production of phytohormones (Spaepen, 2015), the solubilization of insoluble phosphorus into solution available for plant use (Oteino et al., 2015), ACC deaminase enzymes, which effectively reduce plant ethylene levels in the root system (Glick, 2014), siderophores to competitively acquire ferric iron (Solanki et al., 2014), antifungal activity against a variety of plant-pathogenic fungi (Köberl et al., 2013), cell wall degrading enzymes and competition for nutrients and niches (Egamberdieva et al., 2011). Recently, a microbiome shift induced by rhizobacteria was identified as a novel mode of action for biocontrol agents (Schmidt et al., 2012; Erlacher et al., 2014).

In our study, we focused on soybean (*Glycine max* L.) as an important grain legume because it is a source of protein, oil, animal feed, and biodiesel in many countries worldwide, with an annual production of 276.4 Mio t^1^. Improved growth and production of soybeans after biochar application have been reported by Suppadit et al. (2012) and Mete et al. (2015) but mechanisms remain mostly unresolved. We hypothesized that improved growth induced by biochar amendment is strongly linked to interactions with root-associated soil microbes because biochar would promote favorable conditions for microbial proliferation in the rhizosphere. Thus, the main objectives of our study were (i) to evaluate the growth of soybean plants in response to the application of different concentrations of biochar and hydochar, and (ii) to reveal whether char incorporation into soil influences interactions between plants and root-associated microbes that are linked with plant fitness.

## Materials and methods

### Plant growth under greenhouse conditions

The soil used for pot experiments was from an experimental arable field under irrigation (V4) operated by the Experimental Field Station of Leibniz Centre for Agricultural Landscape Research (ZALF), Müncheberg, Germany. The selected chemical and physical properties of soil are as follows: clay and fine silt, 7%; coarse and medium silt, 19%; sand, 74%; Corg – 570 mg 100 g^−1^; pH, 6.2; organic C content, 0.55%; total N content, 0.07%; P content, 32.0 mg (100 g soil)^−1^; K content, 1.25 g (100 g soil)^−1^; and Mg content, 0.18 g (100 g soil^−1^).

The three types of char were supplied from the Leibniz-Institut for Agrartechnik Potsdam-Bornim e.V. (ATB) and used for pot experiments (Reibe et al., 2015a,b): (i) pyrolysis biochar from maize (MBC, 600°C for 30 min), (ii) pyrolysis biochar from wood (WBC, 850°C for 30 min), and (iii) hydrochar from maize silage (HTC char, processed by batch-wise hydrothermal carbonization at 210°C and 23 bar for 8 h). The chemical composition of the chars is presented in Table 1.

**Table 1:** **Characterization of chars (Reibe et al., 2015b)**.

**Material**	**DM (%FW)**	**Ash (%DM)**	**C (%DM)**	**N (%DM)**	**P (g/kg FM)**	**K (g/kg FM)**	**pH**	**EC**
HTC-char	47.39	3.19	64.55	2.09	1.02	3.58	5.25	0.30
MBC-char	92.85	18.42	75.16	1.65	5.26	31.12	9.89	3.08
WBC-char	55.09	16.64	77.62	0.72	1.24	7.8	9.35	1.71

*FM, fresh matter; DM, dry matter; HTC, hydrochar; MBC, maize biochar; WBC, wood biochar; EC, electrical conductivity*.

The soil was mixed with crushed chars (particle size < 3 mm) at increasing rates of 1, 2, and 3% (w/v) just before planting pre-germinated soybean seeds. All pots were arranged in a randomized block design. The soybean seeds (*Glycine max*. cv. Sultana, Naturland Markt, Berlin, Germany) were surface-sterilized using 10% v/v NaOCl for 5 min and 70% ethanol for 5 min, and then rinsed five times with sterile distilled water. Surface-sterilized seeds were transferred on paper tissue soaked in 0.5 mM CaSO_4_ and germinated for 5 days in a dark room at 25°C. The germinated seeds were transferred to pots containing 800 g of soil with four replicates. The treatments were control plants without biochar, soil amended with biochar (MBC, WBC) and HTC char at rates of 1, 2, and 3% (w/v). The plants were grown under greenhouse conditions (day/night temperature 24°C/16°C; humidity 50–60%; day length 12 h) and were watered when necessary. After 6 weeks, the plants were harvested, the roots were separated from shoots and the dry weight was determined.

### Isolation of rhizosphere bacteria

Among the biochar types under study, HTC char showed stimulatory effects on soybean plants in previous experiments and thus was used for further study. Three plants from each treatment, soil without biochar and soil amended with HTC char (2%) were collected for bacterial isolation. Excess soil was removed from the root by shaking, and only tightly adherent soil remained for study. The root samples (10 g for each treatment) were added to 100 ml of PBS buffer (PBS; 20 mM sodium phosphate, 150 mM NaCl, pH 7.0) supplemented with cycloheximide (Sigma, St. Louis, USA) at a final concentration of 100 μg ml ^−1^ and were shaken for 1 h. Serial dilutions (up to 10^−3^) were prepared, and 100 μl from appropriate dilutions was dispensed on Tryptic Soy Agar (TSA, Difco Laboratories, Detroit, USA) for bacterial culture and Peptone dextrose agar (PDA, Difco Laboratories, Detroit, USA) for fungal culture. The plates were incubated at 28°C for 2 days, and the total numbers of bacteria and fungi were counted. The colonies of bacteria that displayed differentiable colony morphologies were picked from plates and were re-streaked on fresh agar plates for purification. One hundred bacterial cultures were selected from each treatment and maintained at 4°C for further study.

### Plant growth stimulation

To test whether bacterial isolates were capable of stimulating plant growth, a pot experiment was conducted in the greenhouse using loamy sand. The seeds were surface-sterilized and inoculated with bacterial strains as described above. The sterility of the seeds was previously tested on TSA agar by incubating the plates for 3 days at 28°C. No contaminants were found, indicating that the surface-sterilization was effective. Two hundred bacterial strains isolated from the rhizosphere of soybeans were grown overnight in Tryptic Soy Broth (TSB), and one milliliter of each culture was pelleted by centrifugation (10,000 × g for 10 min); the supernatant was discarded. Non-inoculated plants were used as negative controls. Cell pellets were washed with 1 ml of PBS and re-suspended in PBS. Cell suspensions corresponded to a cell density of 10^7^ cells/ml. Germinated seeds were placed in the bacterial suspension with sterile forceps and shaken gently. After approximately 10 min, the inoculated seeds were aseptically planted into the potting soil. Three seeds were sown per plastic pot (12 cm diameter; 10 cm deep) to a depth of approximately 1.5 cm. After germination, plants were thinned to one per pot, and pots were set-up in a randomized design with six replications. The plants were grown under greenhouse conditions (day temperature 24°C/night 16°C; humidity 50–60%, day length 12 h) for 1 month. At harvest, the plants were removed from the pots, and the dry weights of roots and shoots were determined. A total 32 strains from control soil and 43 strains from HTC char amended soil were selected based on their plant growth promoting abilities and were further identified and characterized.

### Identification of beneficial plant strains

The identification of isolated strains was performed using whole-cell matrix assisted laser desorption/ionization (MALDI)–time of flight (TOF) mass spectrometry. Sample preparation was carried out according to the ethanol/formic acid extraction protocol recommended by Bruker Daltonics (Bremen, Germany). The isolates were cultured on tryptic soy agar (TSA, Difco Laboratories, Detroit, Michigan, USA) for 24 h, and approximately 10 mg of cell mass was suspended in 300 μL of water and vortexed to generate a homogenous suspension. The suspension was mixed with 900 μL of ethanol and centrifuged. The pellet was resuspended in 50 μL of 70% formic acid and subsequently carefully mixed with 50 μL of acetonitrile. After centrifugation, aliquots of 1 μL of supernatant were placed immediately on spots of a MALDI target. Each spot was allowed to dry and subsequently overlaid with 1 μL of matrix (α-ciano-4-hydroxycinnamic acid in 50% aqueous acetonitrile containing 2.5% trifluoroacetic acid). Mass spectra were acquired using a MALDI-TOF MS spectrometer in a linear positive mode (Microflex™LT, Bruker Daltonics, Bermen, Germany) in a mass range of 2–20 kDa. A bacterial test standard (BTS, Bruker Daltonics, Bremen, Germany) was used for instrument calibration. The raw spectra were imported into MALDI Biotyper™ software (Bruker Daltonics, Germany) and then processed and analyzed using standard pattern matching against the reference spectra in the MALDI Biotyper™ reference database (version 3.0, Bruker Daltonics, Germany).

### *In vitro* screening of bacterial isolates for their PGP activities

#### Indole 3-acetic acid production

Production of IAA (indole 3-acetic acid) was determined as described by Bano and Musarrat (2003). Briefly, bacterial strains were grown in TSB medium. After 3 days, 1 ml of each culture was pelleted by centrifugation, and the supernatant was discarded. Cell pellets were washed with 1 ml of PBS and re-suspended in PBS. One milliliter of cell suspension (corresponding to a cell density of 10^7^cells/ml) was added to 10 ml of TSB amended with tryptophan (100 μg/ml). After 3 days of cultivation, 2 ml aliquots of bacterial cultures were centrifuged at 13.000 × g for 10 min. One milliliter of supernatant was transferred to a fresh tube to which 100 μg/ml of 10 mM orthophosphoric acid and 2 ml of reagent (1 ml of 0.5 M FeCl_3_ in 50 ml of 35% HClO_4_) were added. After 25 min, the absorbance of the developed pink color was read at 530 nm. The IAA concentration in culture was calculated using a calibration curve of pure IAA as a standard.

#### Phosphate solubilization

The phosphate-solubilizing activity of the bacterial strains was determined on Pikovskaya agar (Pikovskaya, 1948) containing precipitated tricalcium phosphate. The bacterial culture grown in TSA medium for 2 days was streaked on the surface of Pikovskaya agar plates and incubated for 3 days. The presence of a clearing zone around bacterial colonies was considered to be an indicator of positive P-solubilization.

#### Production of cell wall degrading enzymes

The cellulose-degrading ability of bacterial isolates was analyzed by streaking inocula on cellulose Congo-Red agar media, as described by Gupta et al. (2012). Zones of clearance around and beneath the colony were detected, indicating enzymatic degradation of cellulose. Lipase activity of the bacterial strains was determined by the Tween lipase indicator assay. Bacterial strains were grown in LA (Luria Agar) containing 2% Tween 80 at 28°C (Howe and Ward, 1976). Protease activity was determined using 5% skimmed milk agar (Brown and Foster, 1970), and pectinase activity was determined using 0.5% pectine amended in M9 medium agar (Kumar et al., 2005).

#### HCN production

For testing HCN production by bacterial strains, the isolates were grown in Kings' B agar medium (KB). A sterilized filter paper saturated with a 1% solution of picric acid and 2% sodium carbonate was placed in the upper lid of the Petri plate. The Petri plate was sealed with Parafilm® M and incubated at 28°C for 3 days. The change in the paper color from yellow to dark blue was recorded as an index of HCN production (Castric, 1975).

#### *In vitro* antibiosis assay

The bacterial isolates were tested *in vitro* for their antagonistic activities against the pathogenic fungi *Fusarium solani, F. culmorum, F. graminearum, Alternaria infectoria*, and *A. teniussima*. The bacterial isolates were grown in TSB broth for 3 days and 50 μL of bacterial culture was dropped into the hole of a PDA plate (4 mm in diameter). Fungal strains were grown in peptone dextrose agar (PDA) plates at 28°C for 5 days, and disks of fresh fungal culture (5 mm diameter) were cut out and placed 2 cm from the hole filled with bacterial filtrate. The plates were sealed with Parafilm® M and incubated at 28°C in darkness until the fungi had grown over the control plates without bacteria. Antifungal activity was recorded as the width of the zone of growth inhibition between the fungus and the test bacterium.

### Statistical analyses

Data were tested for statistical significance using the analysis of variance package included in Microsoft Excel 2007. Comparisons were performed using Student's *t*-test. Mean comparisons were conducted using a least significant difference (LSD) test (*P* = 0.05).

## Results

### Response of soybeans to the type and concentration of biochar

**Figure 1:**
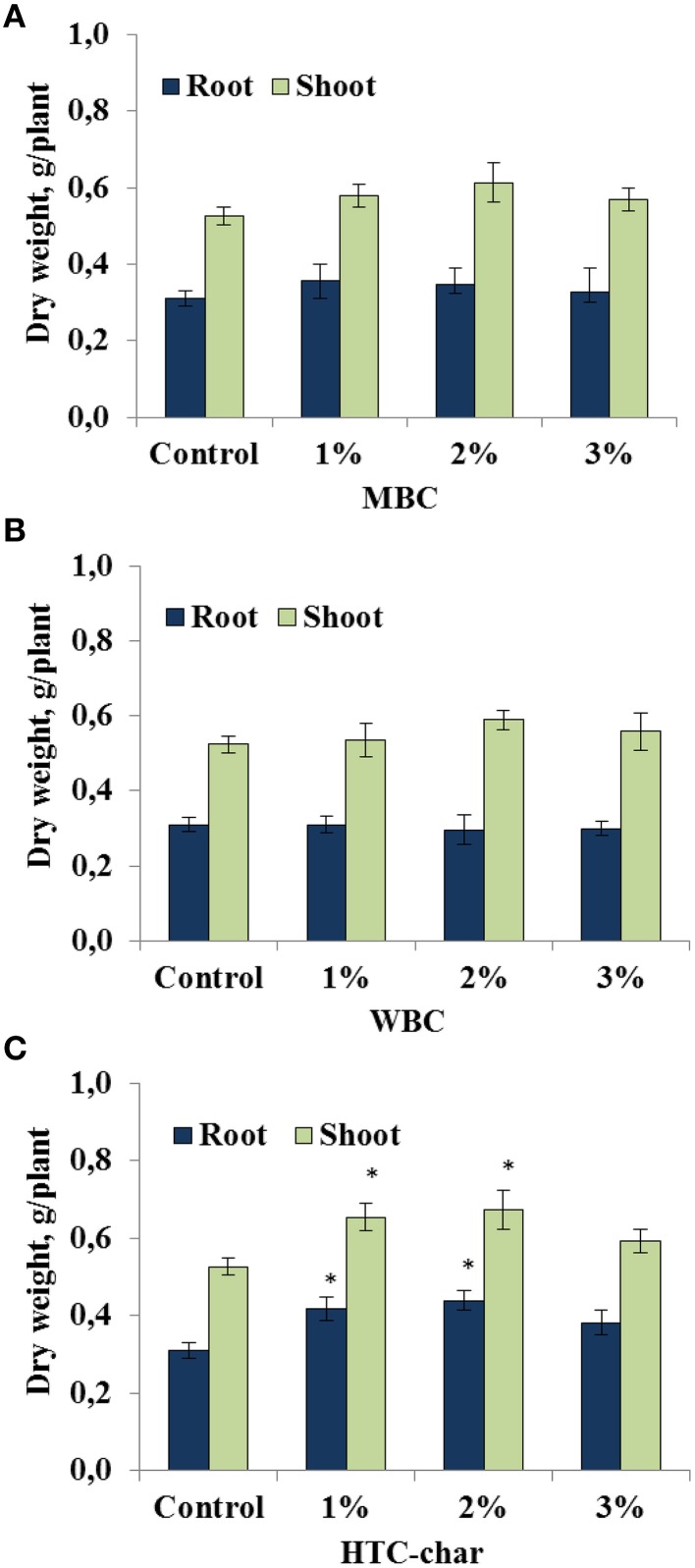
**Root and shoot dry weights of soybeans grown in a greenhouse for 30 days under three maize biochar (MBC) (A), wood biochar (WBC) (B), and HTC-char (C) concentrations (1, 2, and 3%)**. Columns represent means for six plants (*N* = 6) with error bars showing the standard deviation. Columns marked with an asterisk differed significantly from uninoculated plants at *P* < 0.05.

The response of the soybeans to the type of biochar and to different concentrations was investigated under greenhouse conditions. Our study showed that shoot and root biomass of soybeans were not significantly affected by either MBC or WBC amendments in all concentrations (1, 2, and 3%; Figures 1A,B). However, there was a slight but not significant increase in shoot and root growth in the soybeans grown in soil amended with MBC at 1 and 2% concentrations compared with control plants (Figure 1A). In contrast, the root dry weight of soybeans was significantly increased up to 34–41%, and the shoot dry weight was increased up to 24–28% by HTC char amendment at 1 and 2% concentrations, respectively (Figure 1C).

### Enumeration of microbes and isolation of root-associated bacteria

The results of the pot experiments showed that HTC char at a concentration of 2% stimulated the growth of soybeans and thus was used for the characterization of root-associated plant growth promoting bacteria. The bacteria were enumerated after 48 h in the plate count agar and fungi after 5 days in PDA medium. The total numbers of cultivable bacteria isolated from the rhizosphere of plants grown in soil without biochar were 1.5 × 10^7^ CFU (colony-forming units, per gram fresh weight) and 5.3 × 10^7^ CFU (per gram fresh weight) in soil with 2% HTC char. Furthermore, a remarkably greater number of fungi (1.8 × 10^4^ CFU per gram fresh weight) were observed in the rhizosphere of the plants grown in soil without biochar compared with the plants grown in soil amended with 2% HTC char (0.9 × 10^4^ CFU per gram fresh weight).

In total, 200 bacterial strains were isolated from the rhizosphere of soybeans. Among these, 90 isolates were selected from plants grown in control soil, and 110 isolates, from plants grown in HTC char amended soil. All strains were tested for their abilities to stimulate root and shoot growth of soybeans under greenhouse conditions in loamy sand soil. Root and shoot growth stimulating abilities (>20%) were observed in 27–32% of isolates from plants grown in soil without biochar and in 45–57% of isolates from soil amended with 2% HTC char, respectively. A total of 32 isolates from the control plants and 43 isolates from the HTC amended soil induced stimulatory effects on plant growth compared with the non-treated control plants.

### Identification of plant growth promoting bacteria by MALDI-TOF MS

**Figure 2:**
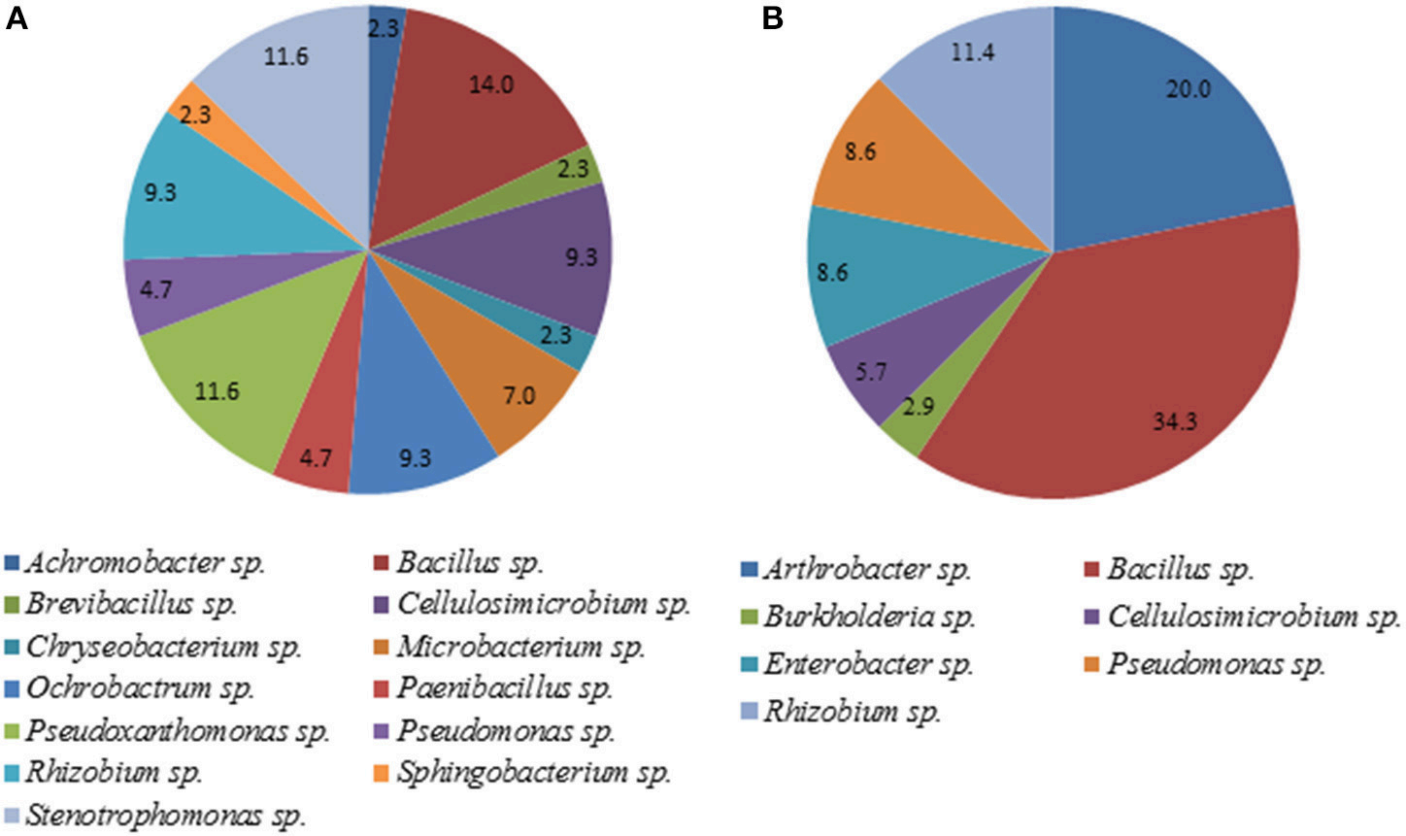
**Diversity of cultivable bacteria in the rhizosphere of soybeans grown in control soil (without char) (A) and HTC-char amended soil (B)**. Numbers indicate the relative abundance, expressed as a percentage of the total number of isolates.

A total of 35 pure isolates from the rhizosphere of control plants and 43 isolates from the rhizosphere of soybeans grown in HTC char amended soil showing plant growth stimulation ability were taxonomically analyzed by MALDI-TOF MS. As shown in Tables 2A,B and Figure 2, there are considerable differences in the diversity of strains isolated from the HTC char amended soil and the control soil. In the rhizosphere of soybeans grown in the control soil, isolates were affiliated with seven genera, whereas 24 isolates were identified at the species level. *Bacillus* was the predominant genus, which was followed by the genera *Arthrobacter* and *Rhizobium*. Furthermore, isolates affiliated with the genera *Cellulosimicrobium, Enterobacter* and *Pseudomonas* were also found. The most abundant species were identified as *Rhizobium radiobacter* (C14, C53, C19, C87), followed by the species *Arthrobacter globiformis* (C3, C16), *Bacillus megaterium* (C32, C38), *Cellulosimicrobium cellulans* (C29, C42), *Enterobacter asburiae* (C46, C50), and *Pseudomonas chlororaphis* (C28, C44). Only one isolate was identified as *Burkholderia terricola* (Figure 2).

**Table 2A:** **Plant growth promoting traits of strains isolated from the rhizosphere of soybeans grown in soil without hydrochar**.

**Isolate number**	**Identity according to MALDI Biotyper**		**Antagonistic activity (zone of inhibition, mm)**					**Production of exo-enzymes**						
		Score^*^	***F. solani***	***F. culmorum***	***F. graminearum***	***A. infectoria***	***A. teniussima***	**Lipase**	**Protease**	**Pectinase**	**Cellulase**	**IAA synthesis**	**P- solubilization**	**HCN**
C5	*Arthrobacter* sp.	+	−	−	−	−	−	−	−	−	−	−	+	−
C9	*Arthrobacter* sp.	+	−	−	−	−	−	−	+	−	−	−	−	−
C99	*Arthrobacter oxydans*	++	−	−	−	−	−	−	−	−	−	+	+	−
C3	*Arthrobacter globiformis*	+++	−	−	−	−	−	−	−	−	−	−	−	−
C16	*Arthrobacter globiformis*	++	−	−	−	−	−	−	−	+	−	+	−	−
C41	*Arthrobacter histidinolovorans*	++	−	−	−	−	−	−	−	−	+	−	−	−
C71	*Arthrobacter* sp.	+	−	−	−	−	−	−	−	−	+	+	−	−
C13	*Bacillus* sp.	+	−	−	−	−	−	−	−	−	−	−	−	−
C18	*Bacillus* sp.	+	−	−	−	−	−	−	−	−	+	−	−	−
C79	*Bacillus altitudinis*	++	−	−	−	−	−	−	−	+	−	−	+	−
C75	*Bacillus* sp.	+	−	−	−	−	−	+	−	+	−	+	−	−
C21	*Bacillus* sp.	+	−	−	−	−	−	−	−	−	−	−	−	−
C20	*Bacillus* sp.	+	−	−	−	−	−	−	−	−	+	−	−	−
C49	*Bacillus pumilus*	++	−	−	−	+^2^	+^2^	−	−	+	−	−	−	+
C30	*Bacillus licheniformis*	++	−	−	−	−	−	−	−	−	−	+	−	−
C32	*Bacillus megaterium*	++	−	−	−	−	−	−	−	−	−	−	+	−
C38	*Bacillus megaterium*	++	−	−	−	−	−	−	−	−	−	+	−	−
C90	*Bacillus cereus*	++	+^4^	+^3^	+^4^	−	−	−	+	−	−	−	−	+
C78	*Bacillus pumilus*	++	−	−	−	−	−	−	−	−	+	−	−	+
C35	*Burkholderia terricola*	++	−	−	−	−	−	−	−	−	−	−	−	−
C29	*Cellulosimicrobium cellulans*	++	−	−	+^3^	−	−	−	−	−	−	−	−	−
C42	*Cellulosimicrobium cellulans*	++	−	+^4^	−	−	−	−	−	−	+	−	−	−
C33	*Enterobacter cloacae*	+++	−	+^5^	−	−	−	−	−	−	−	+	+	−
C46	*Enterobacter asburiae*	++	−	−	−	−	−	−	−	−	+	−	−	−
C50	*Enterobacter asburiae*	++	+^8^	+^5^	−	−	+^4^	−	+	−	−	−	−	−
C27	*Pseudomonas putida*	++	−	+^10^	+^12^	−	−	−	−	+	+	+	−	−
C28	*Pseudomonas chlororaphis*	++	+^5^	+^4^	+^5^	−	+^3^	+	−	−	+	−	−	−
C44	*Pseudomonas chlororaphis*	++	−	−	−	−	−	−	+	−	−	+	−	−
C14	*Rhizobium radiobacter*	+++	−	−	−	−	−	−	−	−	−	−	+	−
C53	*Rhizobium radiobacter*	+++	−	−	−	−	−	−	−	−	+	−	−	−
C19	*Rhizobium radiobacter*	++	−	−	−	+^2^	+^2^	−	−	+	−	−	−	+
C87	*Rhizobium radiobacter*	++	−	+^3^	−	−	−	−	−	+	−	+	−	−
C40	No reliable identification		−	+^3^	−	+^3^	−	−	−	−	−	−	−	−
C6	No reliable identification		−	+^3^	−	+^3^	−	−	−	−	−	−	+	−
C24	No reliable identification		−	−	−	−	−	−	−	−	−	−	−	−

* *+++, highly probable species identification; ++, secure genus identification; +, probable genus identification*.

**Table 2B:** **Plant growth promoting traits of strains isolated from the rhizosphere of soybeans grown in soil amended with 2% HTC char**.

**Isolate number**	**Identity according to MALD Biotyper**		**Antagonistic activity (zone of inhibition, mm)**					**Production of exo-enzymes**						
		Score^*^	***F. solani***	***F. culmorum***	***F. graminearum***	***A. infectoria***	***A. teniussima***	**Lipase**	**Protease**	**Pectinase**	**Cellulase**	**IAA synthesis**	**P- solubilization**	**HCN**
H58	*Achromobacter* sp.	+	−	−	−	−	−	−	+	−	−	+	−	−
H53	*Bacillus* sp.	+	−	+^4^	+^5^	−	−	−	−	−	+	+	−	+
H2	*Bacillus* sp.	+	−	−	−	−	−	−	−	−	+	−	+	−
H82	*Bacillus megaterium*	++	−	+^6^	+^8^	−	−	−	+	+	−	+	−	−
H61	*Bacillus* sp.	+	−	−	−	−	−	−	−	+	−	+	+	−
H28	*Bacillus* sp.	+	−	−	−	+^4^	+^6^	−	+	+	+	−	−	−
H67	*Bacillus simplex*	++	−	−	−	−	−	+	+	−	−	−	−	−
H22	*Brevibacillus laterosporus*	+++	−	−	−	+^4^	+^4^	−	−	+	−	+	+	−
H4	*Cellulosimicrobium cellulans*	++	+^6^	−	−	−	−	−	−	−	−	−	+	−
H90	*Cellulosimicrobium cellulans*	++	−	+^8^	+^6^	+^4^	+^4^	−	−	−	−	−	−	−
H12	*Cellulosimicrobium cellulans*	++	−	−	−	−	−	+	−	+	−	−	−	−
H20	*Cellulosimicrobium cellulans*	++	+^4^	−	−	−	−	−	−	−	−	+	+	−
H63	*Chryseobacterium* sp.	+	−	−	−	−	−	−	+	−	+	−	+	−
H101	*Microbacterium natoriense*	+++	−	−	−	−	−	−	−	−	−	+	−	−
H58c	*Microbacterium* sp.	+	−	+^4^	+^6^	−	−	−	−	+	+	−	−	+
H84c	*M. trichothecenolyticum*	+++	−	−	−	−	−	−	−	−	−	−	+	−
H7	*Ochrobactrum intermedium*	+++	−	−	−	−	−	−	−	−	+	+	−	−
H26	*Ochrobactrum intermedium*	+++	−	−	−	−	−	−	+	−	−	−	−	−
H86	*Ochrobactrum intermedium*	+++	−	−	−	−	−	−	−	−	+	−	−	−
H65	*Ochrobactrum intermedium*	+++	−	−	−	−	−	−	−	+	−	−	−	−
H44	*Paenibacillus polymyxa*	+++	−	+^6^	+^6^	+^8^	−	−	−	+	+	+	+	−
H78	*Paenibacillus* sp.	+	−	+^4^	+^4^	+^6^	−	−	+	−	+	+	+	−
H31	*Pseudoxanthomonas kaohsiungensis*	+++	−	−	−	+^6^	−	−	−	+	−	+	+	−
H37	*Pseudoxanthomonas kaohsiungensis*	++	−	−	−	−	−	+	+	+	+	−	−	−
H55	*Pseudoxanthomonas kaohsiungensis*	+++	−	−	−	−	−	−	−	−	−	−	−	−
H79	*Pseudoxanthomonas kaohsiungensis*	+++	−	−	−	−	−	−	−	−	−	−	−	−
H100	*Pseudoxanthomonas kaohsiungensis*	+++	−	−	−	−	−	−	−	−	−	−	−	−
H70	*Pseudomonas putida*	+++	−	−	−	−	−	−	−	+	−	+	+	−
H73	*Pseudomonas putida*	+++	−	−	+^6^	+^8^	−	−	−	+	−	+	+	−
H1	*Rhizobium radiobacter*	++	−	−	−	−	−	−	+	+	−	−	−	−
H8	*Rhizobium radiobacter*	++	+^4^	+^6^	−	−	−	+	−	−	−	+	−	−
H14	*Rhizobium radiobacter*	+++	−	+^5^	−	−	−	−	+	−	−	−	−	+
H76	*Rhizobium radiobacter*	++	+^6^	+^4^	+^4^	−	−	−	−	−	−	+	+	+
H72	*Sphingobacterium* sp.	+	−	+^4^	−	−	−	−	−	−	−	+	+	−
H69	*Stenotrophomonas* sp.	+	−	−	−	−	−	−	+	+	−	−	−	−
H93	*Stenotrophomonas* sp.	++	−	−	−	−	−	−	−	−	−	+	−	−
H92	*Stenotrophomonas* sp.	++	+^6^	+^10^	+^6^	−	−	−	−	−	−	−	+	−
H75	*Stenotrophomonas* sp.	+	+^2^	+^2^	+^4^	−	−	+	+	+	+	+	−	+
H66	*Stenotrophomonas maltophilia*	++	−	+^14^	+^12^	−	−	+	+	+	+	+	−	+
H3	No reliable identification		−	+^8^	−	−	−	−	+	+	−	−	+	−
H6	No reliable identification		−	−	−	−	−	−	−	−	−	−	−	+
H17	No reliable identification		−	−	−	+^4^	−	−	−	−	−	−	−	−
H56	No reliable identification		−	−	−	−	−	−	−	−	−	+	−	−

* *+++, highly probable species identification; ++, secure genus identification; +, probable genus identification*.

A total of 13 bacterial genera were isolated from the rhizosphere of soybeans grown in HTC char amended soil, whereas 12 isolates were identified at the species level (Table 2B). The isolates from biochar amended soil showed a greater diversity compared with the isolates originating from the plant rhizosphere of the control soil. The most abundant isolates were identified as *Cellulosimicrobium cellulans* (H4, H90, H12, H20), *Ochrobactrum intermedium* (H7, H26, H86, H65), *Pseudoxanthomonas kaohsiungensis* (H31, H37, H55, H79, H100), and *Stenotrophomonas* sp. (H69, H93, H92, H75). Members of the genera *Achromobacter, Brevibacillus, Chryseobacterium, Microbacterium, Ochrobactrum, Paenibacillus, Pseudoxanthomonas, Sphingobacterium* and *Stenotrophomonas* were not found among isolates from the control soil.

### *In vitro* plant growth promoting traits

All bacterial strains isolated from the rhizosphere of soybeans grown in HTC char amended soil and without biochar were screened for multiple plant growth promoting traits. Most of the bacterial isolates exhibited one or more plant growth-promoting activities (Tables 2A,B).

The production of the phytohormone IAA by bacterial isolates is shown in Table 2A. A large amount of the rhizosphere isolates (48%) from HTC char amended soil produced IAA, whereas only 28% of the isolates from control soil showed IAA production. Most of the IAA producing isolates from control soil belonged to the genera *Arthrobacter* (C99, C16, C71) and *Bacillus* (C21, C32, C90). Three isolates belonging to the genus *Stenotrophomonas* (H93, H75, H66) from HTC char amended soil showed IAA activity, followed by the genera *Cellulosimicrobium* (H90, H20), *Pseudomonas* (H70, H73) and *Rhizobium* (H8, H76).

Positive P-solubilization was observed in 7 strains from 4 genera (20%) originating from plants grown in control soil and 16 strains from 11 genera (37%) originating from HTC char amended soil. All bacterial isolates were screened for their ability to suppress plant pathogenic fungi, such as *Fusarium solani, F. culmorum, F. graminearum, Alternaria infectoria*, and *A. teniussima*. The proportions of isolates with antagonistic activity to one or more pathogens was higher for the HTC char amended soil (51%) than for the control soil (28%). As shown in Table 2A, two *Pseudomonas chlororaphis* strains, C27 and C28, from control soil and six isolates, *Stenotrophomonas maltophilia* H66, *Stenotrophomonas* sp. H92*, Cellulosimicrobium cellulans* H90, *Bacillus megaterium* H82*, Paenibacillus polymyxa* C44, *and Pseudomonas putida* 73, from HTC char amended soil exerted the highest inhibition of mycelial growth of the genus *Fusarium*.

The ability of isolates to produce cell wall degrading enzymes, as well as proteases and lipases, was also determined. The isolates from the HTC char amended soil exhibited a higher proportion of enzyme producers than the control soil, where lipase, protease, pectinase and cellulase activity were detected in 14, 33, 40, and 26% of the isolates, respectively. The percentage of enzyme producing bacteria isolated from control soil was lower, where only 6% of isolates exhibited lipase, 11% protease, 20% pectinase, and 29% cellulase activity. Out of isolates that exhibited plant growth-promoting activities *in vitro*, eight isolates (H66, H75, H72, H76, H73, H44, H22, and H90) originating from HTC char amended soil and six isolates (C99, C28, C46, C78, C30, and C87) originating from control soil were selected for plant growth stimulation under greenhouse conditions.

### Plant growth stimulation

**Figure 3:**
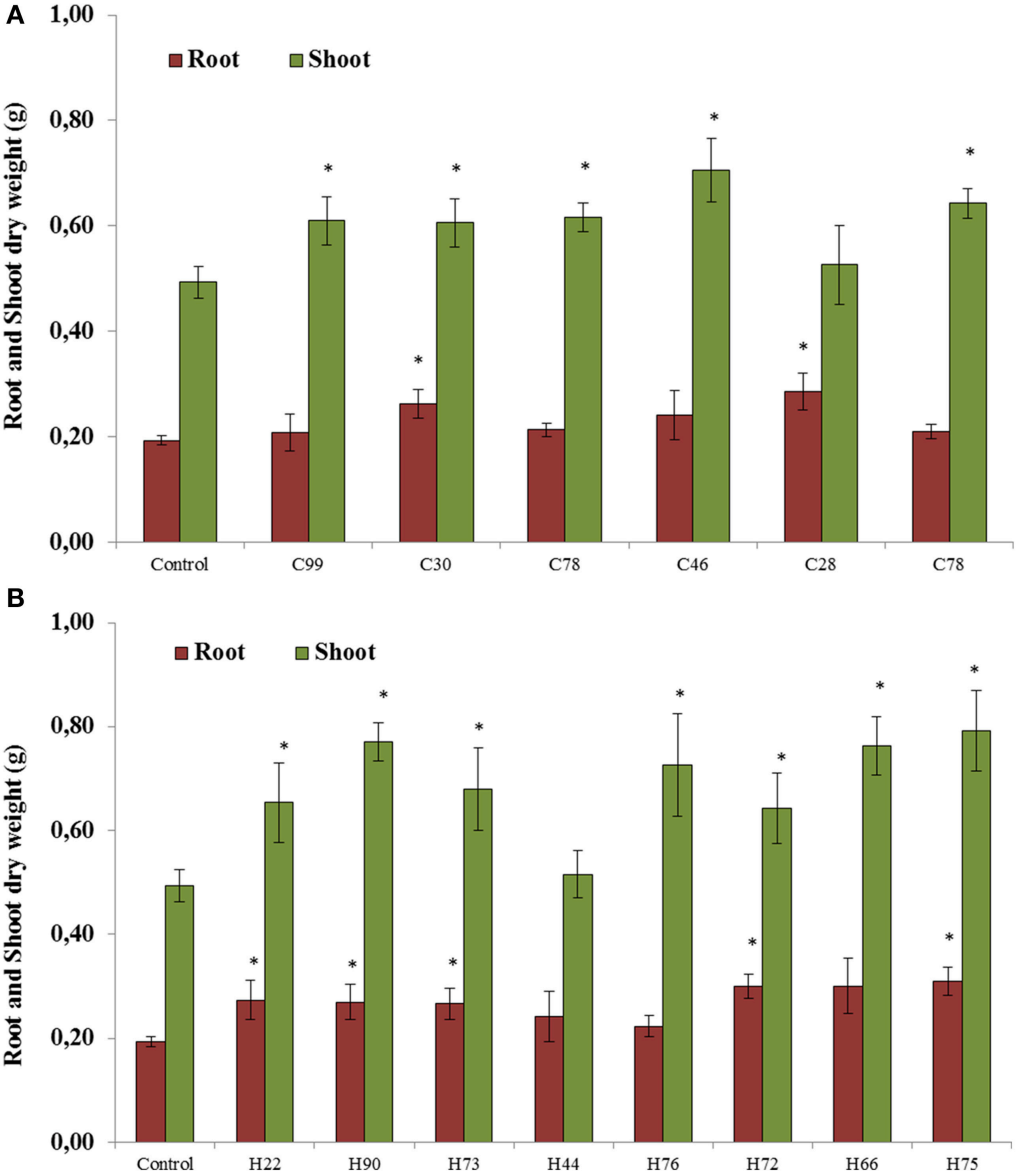
**Root and shoot dry weight of soybeans when seedlings were inoculated with bacterial strains isolated from soybeans grown in soil without char (A) and in 2% HTC-char (B) amended soil**. Columns represent the means for six plants (*N* = 6), with error bars showing the standard deviation. Columns marked with an asterisk differed significantly from uninoculated plants at *P* < 0.05.

All 14 selected bacterial strains were screened for plant growth stimulating abilities in pots under greenhouse conditions. The results showed that six strains isolated from plants grown in the control soil without biochar significantly increased root or shoot dry weight compared with the untreated controls (Figures 3A,B). The root dry weight increased up to 51% after inoculation with *Pseudomonas chlororaphis* (C28) and the shoot dry weight increased up to 44% with *Enterobacter asburiae* (C46; Figure 3A). Significant increases (between 28 and 63%) in plant dry weight relative to non-inoculated controls were observed with isolates from HTC char amended soil. The isolates *Cellulosimicrobium cellulans* (H90), *Pseudomonas putida* (H73), *Stenotrophomonas maltophilia* (H66) and *Stenotrophomonas* sp. (H75) showed significantly higher plant growth stimulation, from 40 to 63% (Figure 3B).

## Discussion

Biochar incorporation into soil has been shown to enhance plant growth, to sequester carbon and to improve soil fertility, and moreover to protect plants from various soil borne pathogens (Lehmann and Joseph, 2009; Zimmerman, 2010). The increase in plant growth with biochar application has been reported for various species such as pine and alder (Robertson et al., 2012), peanut (Agegnehu et al., 2015), tomato (Vaccari et al., 2015), wheat (Akhtar et al., 2015) and also soybean (Sanvong and Nathewet, 2014)—however, several other studies reported no significant effect on plant growth (Chan et al., 2007; Van Zwieten et al., 2010). In summary, these observations indicate that effects of biochar on plant growth depend on the type of biochar, the application rate, and soil properties (Alburquerque et al., 2014) but mechanisms behind effects mostly remain unresolved. In our study, we confirmed a positive impact of HTC char treatment on the growth of soybean, but not in case of either MBC or WBC amendments. Similar observations were reported by Reibe et al. (2015b), when Pyro-char (MBC) and HTC-char applications resulted in significantly higher dry matter yields of wheat after 6 weeks of growth in rhizoboxes, as compared to Pyreq-char (WBC) or a control. There are several possible reasons why hydrochar might increase plant growth and enhance nutrient acquisition. Hyrdochar contains a higher amount of labile carbon fractions (Cao et al., 2010), which may stimulate microbial activity and thereby improve soil nutrient cycling (Kolb et al., 2009). Furthermore, hydrochars were found to reduce nitrogen losses from soil by immobilization and may provide nitrogen in plant-available form (Libra et al., 2011), whereas Pyro-chars contain less nitrogen with a decreased availability to plants (Gaskin et al., 2010).

Furthermore, HTC biochar amendment showed an impact on root associated microbes and on microbial interactions with plants, which were previously rarely studied in this context. Our findings are confirmed by the results of Kolton et al. (2011), who showed a clear shift in the total root-associated microbial community composition of mature sweet pepper (*Capsicum annuum* L.) after amendment with biochar from citrus wood. In our study, the analysis of cultivable root associated bacteria demonstrated that HTC char amendments increased bacterial populations in the rhizosphere of soybeans compared with control plants, whereas fungal growth was decreased over the control, in agreement with Chen et al. (2013). An increased microbial activity in the rhizosphere after the addition of hydrochar could be explained as a result of changes in soil chemical and physical properties in the root surface area. Prendergast-Miller et al. (2014) observed that biochar-amended soils had larger rhizosphere zones than the control. Moreover, the rhizosphere contained biochar particles providing additional labile carbon, nitrogen and phosphorus sources and also habitat niches, supporting bacterial proliferation and persistence in the rhizosphere.

In all rhizosphere samples from soybeans, we found a high diversity of potential plant growth promoting rhizobacteria. However, the species composition in the treated and non-treated plants was different. The most abundant species isolated from soybeans grown in control soil were *Rhizobium radiobacter, Arthrobacter globiformis*, and *Bacillus megaterium*, whereas in HTC char amended soil, *Cellulosimicrobium cellulans, Ochrobactrum intermedium, Pseudoxanthomonas kaohsiungensis*, and *Stenotrophomonas* sp. were dominant. The species identified in our study are already known for their plant growth promoting abilities, e.g., *R. radiobacter* stimulated growth of barley (*Hordeum vulgare*; Humphry et al., 2007), and *B. megaterium* stimulated growth of bean (*Phaseolus vulgaris*; Ortíz-Castro et al., 2005). Furthermore, a strain of the species *C. cellulans* (KUGr3) is able to form IAA, solubilize phosphate and stimulated growth of chili plants (*Capsicum annuam*; Chatterjee et al., 2009). *O. intermedium* increased seed germination, root and shoot length, and grain yield in lentil (*Lens esculenta*; Faisal, 2013). Several *Stenotrophomonas* sp. strains increased root and shoot growth and the nutrient uptake of soybean (*Glycine max*), cucumber (*Cucumis sativus*), and tomato (*Solanum lycopersicum*; Egamberdieva et al., 2011; Berg and Martinez, 2015).

In addition, the beneficial properties of species in treated and non-treated plants were different. Compared with control soil, a higher proportion of isolates from the HTC char amended soil was found to produce IAA, HCN and cell wall degrading enzymes. Furthermore, a higher proportion of bacterial isolates was capable of hydrolyzing organic and inorganic phosphorus from insoluble compounds and showed antagonistic activity to one or more pathogens. In the present study, a decrease in fungal populations (~50% reduction) was observed after HTC char addition. The increased proportion of bacteria capable of inhibiting fungal pathogens following amendment of HTC char suggests that the observed suppression of the fungal population was due to antagonistic interactions of microbes. The phytohormone IAA is a naturally occurring auxin which has a major role in the regulation of plant growth. The stimulation of the growth of various plants by inoculation with PGPR and IAA producing ability is well-documented (Egamberdieva, 2009, 2012; Berg et al., 2010). Phytohormones produced by root-associated bacteria will be taken up by plant cells, stimulate cell proliferation, and enlarge the root system so that nutrients and water can be taken up more efficiently. For example, IAA producing *Stenotrophomonas rhizophila* significantly affected plant growth, N and P uptake and the number of nodules in soybean (Egamberdieva et al., 2015). Similarly, multiple isolates from the rhizosphere that suppress fungal growth by the production of HCN, cell wall degrading enzymes or antifungal compounds were used to prevent and control fungal diseases (Berg et al., 2013b; Maurer et al., 2013). Seed coating with *Pseudomonas* strains antagonistic to soilborne pathogens, such as *Sclerotium rolfsii, Fusarium oxysporum*, and *Rhizoctonia solani*, produced siderophores, chitinase, and HCN and were therefore able to suppress infections in soybean seedlings by fungal pathogens (Susilowati et al., 2011). In another study, the charcoal root rot of soybean caused by *Macrophomina phaseolina* was attenuated by the antagonistic bacterial strains *P. agglomerans* and *Bacillus* sp. under greenhouse conditions (Vasebi et al., 2013). The mechanisms involved in plant growth stimulation and the biological control of plant pathogens were also observed for bacterial isolates in our study and were thus further evaluated for their impact on plant growth promotion of soybeans under greenhouse conditions. Indeed, inoculation of soybeans with these isolates led to significant increases in plant growth and development. In previous studies, PGPR *Stenotrophomonas rhizophila* was able to stimulate root and shoot growth, nodulation and nutrient uptake of soybeans under greenhouse conditions (Egamberdieva et al., 2015). Similarly, Aung et al. (2013) found a significant increase in shoot and root biomass, as well as nodulation in soybeans inoculated with *Azospirillum* sp., compared to non-inoculated controls under pot conditions.

From our study, we conclude that increased plant growth in response to soil amendment with biochar is based on the type of char, i.e., HTC application increased growth of soybean but not in case of either MBC or WBC. Moreover, HTC application was shown to alter the community composition of root associated microbes exhibiting plant growth-promoting activities *in vitro* such as phytohormone production and suppression of fungal pathogens. Thus, we provided evidence that improved plant growth by hydrochar incorporation into soil is mostly an indirect rather than a direct effect that depends on the type of char and the activity of plant-associated beneficial soil bacteria. The stimulation of certain plant-beneficial bacteria by biochar also suggests the possibility of developing combined approaches of biochar treatment and biological control solutions (Berg et al., 2013b).

## Author contributions

DE, SW, and GB did experimental design work. DE and UB conducted experiments. EA analyzed the data. DE, SW, and GB wrote the manuscript. All authors read and approved the manuscript.

### Conflict of interest statement

The authors declare that the research was conducted in the absence of any commercial or financial relationships that could be construed as a potential conflict of interest.

## Footnotes

^1^FAOstat (2013). Available online at: http://faostat.fao.org/ (2013-02-25).
